# Proteomic insights into intra- and intercellular plant-bacteria symbiotic association during root nodule formation

**DOI:** 10.3389/fpls.2013.00028

**Published:** 2013-02-25

**Authors:** Afshin Salavati, Alireza Shafeinia, Katarina Klubicova, Ali A. S. Bushehri, Setsuko Komatsu

**Affiliations:** ^1^Department of Plant Breeding and Biotechnology, Ramin University of Agriculture and Natural ResourcesAhwaz, Iran; ^2^Institute of Plant Genetics and Biotechnology, Slovak Academy of SciencesNitra, Slovakia; ^3^Department of Agronomy and Plant Breeding, College of Agriculture and Natural Resources, University of TehranKaraj, Iran; ^4^National Institute of Crop Science, National Agriculture and Food Research OrganizationTsukuba, Japan

**Keywords:** proteomics, legumes, bacteria, symbiosis, nodule development

## Abstract

Over the last several decades, there have been a large number of studies done on the all aspects of legumes and bacteria which participate in nitrogen-fixing symbiosis. The analysis of legume–bacteria interaction is not just a matter of numerical complexity in terms of variants of gene products that can arise from a single gene. Bacteria regulate their quorum-sensing genes to enhance their ability to induce conjugation of plasmids and symbiotic islands, and various protein secretion mechanisms; that can stimulate a collection of chain reactions including species-specific combinations of plant-secretion isoflavonoids, complicated calcium signaling pathways and autoregulation of nodulation mechanisms. Quorum-sensing systems are introduced by the intra- and intercellular organization of gene products lead to protein–protein interactions or targeting of proteins to specific cellular structures. In this study, an attempt has been made to review significant contributions related to nodule formation and development and their impacts on cell proteome for better understanding of plant–bacterium interaction mechanism at protein level. This review would not only provide new insights into the plant–bacteria symbiosis response mechanisms but would also highlights the importance of studying changes in protein abundance inside and outside of cells in response to symbiosis. Furthermore, the application to agriculture program of plant–bacteria interaction will be discussed.

## INTRODUCTION

Mutualistic symbiosis includes a wide range of interactions among a diverse set of organisms. The symbiosis between legumes and rhizobia is a classic mutualistic relationship and nitrogen fixation process is one of the most important biological processes on the earth. The symbiosis culminates in the creation of a highly specialized plant organ, the root nodule, with plant cells invaded by bacteria. More than a century ago, Hellriegel and Wilfarth (1888) identified rhizobia as a source of nitrogen fixation. Rhizobia, Gram-negative soil bacteria, induce the formation of nodules in many, but not all, leguminous plants (Gualtieri and Bisseling, 2000). The nitrogen fixing nodule is a model for plant developmental processes and plant–microbe interactions. The nodule forms an anaerobic niche for nitrogen fixation, protecting the bacterial nitrogenase from inactivation by O_2_. In exchange for carbohydrates provided by the host legume, fixed nitrogen would be supplied by bacteria to the legume.

Establishment of symbiosis between host plants and symbiotic bacteria is a multistage process covering signal perception, transduction, and responses (Broughton et al., 2000). These processes depend on the precise spatial and temporal regulation of nod- and other symbiotic genes (Schlaman et al., 1998). Synchronized expression of symbiotic loci in legumes and their bacterial partner involves the exchange of a series of molecular signals allowing rhizobia to invade the plant roots. Rhizobia produce various molecular signals that influence the host plants at various steps along the symbiotic pathway (Perret et al., 2000). Symbiosis is initiated when accumulated flavonoids in the rhizosphere of the host plant prompt the cascade of rhizobial signal transduction by interacting with transcriptional activators of nodulation genes. This flavonoid-modulated signal transduction cascade regulates expression of genes that act during nodule development. Most nodulation genes including nol, noe, and nod, are involved in the synthesis of host-specific lipochitooligosaccharides (Nod factors), which are essential for the initial infection of root hairs (Perret et al., 2000). Nod factors provoke root curling, creation of nodule primordia, early nodulin (*ENOD*) genes expression, and finally allow the bacteria to enter the root hairs (Broughton et al., 2000; Geurts and Bisseling, 2002). Thus, flavonoids and Nod factors correspond to the primary sets of signal transduction by the symbiotic partners.

Further, already differentiated cortical cells have to be reactivated and enter the cell cycle from their arrested state, so that a nodule primordium is formed. Release of the bacteria into primordium cells results in its differentiation into a nodule (Gage, 2004; Oldroyd and Downie, 2004). The infection process as well as the induction of cortical cell divisions is caused by Nod factors that are secreted by rhizobia when they colonize the roots of their host. In legumes, the infection process starts in epidermal root hair cells (Brewin, 2004). Nod factor-secreting rhizobia induce deformations in most of the root hairs in a region of the root that is susceptible to the interaction (Heidstra et al., 1994). In this way, the bacterium becomes entrapped within a three-dimensional cavity of curl and a small colony of rhizobia is formed. Subsequently, these rhizobia induce local weakening of the cell wall and, by invagination of the plasma membrane of the root hair, a tube-like structure is formed. This is the so-called infection thread (IT) that allows the rhizobia to penetrate the root-hair cell. Each root cortical cell that traversed to make an IT and ultimately the rhizobia reach the nodule primordium (Brewin, 2004). Cortical roots taken up in the nodule primordium cells in an endocytosis-like manner forming organelle-like structures (symbiosomes) that contain one or more bacteria, which upon differentiation start to fix nitrogen (Brewin, 2004).

Proteomics is a high-throughput technology that has been used to investigate a wide range of biological aspects including phylogenetic and molecular divergence studies (Larrainzar et al., 2007;) plant responses to different stresses (Larrainzar et al., 2007; Danchenko et al., 2009; Klubicova et al., 2012; Komatsu et al., 2012; Mohammadi et al., 2012; Salavati et al., 2012b) detailed studies on the structural components, and biochemical pathways involved in symbiotic nitrogen fixation (SNF; Broghammer et al., 2012; Navascués et al., 2012; Rose et al., 2012). Approaches such as transcriptome, proteome, and metabolome analysis in both symbionts, promise to reveal much more detail about the metabolic flows in the nitrogen fixing nodule or even to description the novel unknown aspects (Delmotte et al., 2010; Khatoon et al., 2012; Salavati et al., 2012a,c). Several proteomic studies focused on characterization of different aspects and stages of plant–microbe interactions were published. Natera et al. (2000) identified root nodule proteins in* Melilotus alba* during 12 days after inoculation by *Sinorhizobium meliloti*, including *S. meliloti* and bacteroid proteins. Proteins involved in nodule formation and regulated by auxin were identified in *Medicago truncatula* infected by *S. meliloti* (van Noorden et al., 2007). Other previous studies described proteomes of microbial symbionts. Analysis of *hfg* mutant *S. meliloti*, drastically affected in its ability to colonize and initiate symbiosis, showed that ABC type transporter system represents most abundant class of differentially expressed proteins (Barra-Bily et al., 2010). Extracellular proteome of *Rhizobium etli* strain during different growth stages was described by Meneses et al. (2010). Their results suggests that secretome of *R. etli* consists of actively secreted proteins, which mostly are extracellular enzymes (mostly degradation enzymes) and proteins that bind nutrients and extracellular appendages, and proteins that have functions in the cytosol and are not actively secreted but may be released into the culture medium. Function of many identified proteins in extracellular proteome is still unknown.

In addition, the organeller proteins with potential role in the entry of symbiotic bacteria into plant roots or in the other steps of symbiotic processes were also studied (Robertson et al., 1978; Wienkoop and Saalbach, 2003; Hoa et al., 2004; Imaizumi-Anraku et al., 2005). First study focused on phosphoproteome changes during symbiosis was performed by Rose et al. (2012).

Also a few reviews concerning proteomic analysis of plant–microbe interactions in general were published (Rolfe et al., 2003; Bestel-Corre et al., 2004; Mathesius, 2009; Muneer et al., 2012). Our review is more focused to SNF. The integration of genomics and post-genomics events is a strong consensus for functional study of plant–microbe interactions, in general, and SNF, in specific. Model legume genomics and the continued effort on cultivated grain and pasture legumes open unique possibilities for family-based comparative genomics in the Leguminosae. Proteomic studies in combination with transcriptomics studies such as quantitative RT-PCR can advance symbiosis analysis to a new level (Resendis-Antonio et al., 2011; Salavati et al., 2012a). In combination with the on-going genome sequencing and growing EST collection of the model legumes, proteomics has been recently become a powerful investigation of the most detailed physiological events in plant, animal and microorganisms (Colebatch et al., 2004; Thibivilliers et al., 2009). In this paper, we focused on the large-scale identification of proteins and their complexes coupled to genome- and EST-sequence information, which can be used to identify proteins and to monitor changes in protein expression as a function of developmental stages, to review legume nodule initiation and developmental events at translational level.

As technical view, sample preparation is an important step in proteomics researches. This step is principally difficult in studies with plants. Many plant tissues are often rich in proteases contaminants such as polysaccharides, lipids, and phenols (Carpentier et al., 2005). Furthermore, it is necessary to acquire high-quality gels showing reproducible protein patterns (Hurkman and Tanaka, 1986). The extraction method must conserve the quality and quantity of the extracted proteins (Hurkman and Tanaka, 1986; Westermeier and Naven, 2002). Although a single-step process for protein extraction would be highly desirable, no unique sample preparation method can be used to 2-DE analysis (Dunn, 1999). Therefore, many researchers developed and optimized some efficient methods such as a phenol/SDS-based method (Wang et al., 2006; Rodrigues et al., 2012) and non-phenol-based methods (Guerreiro et al., 1997; Natera et al., 2000), to find a simple method that could be applied regularly to proteomics studies of symbiosis interactions.

## OVERVIEW OF RHIZOBIUM–LEGUME INTERACTIONS

### ROOT ATTACHMENT

Among root-derived compounds, some phenolic-based compounds act as chemotactic attractants and, on the other hand, the secreted and surface proteins are involved in rhizobia attachment to root hairs in the initiation step of the symbiosis (Peters and Verma, 1990; Deakin and Broughton, 2009). Although being in the right place at the right time is critical to the instigation of nodulation, the principal aspects of root attachment, including close contiguity to root hairs, clonal events, and root hair curling, have crucial importance. These steps ensure a supply of nutrients that enable the bacteria to grow on and around the root, determine whether they will be the ones that can successfully initiate infection in many legumes (Downie, 2010). The fundamentals of communication between the prospective symbiotic partners were established a few years ago (Downie, 1998; Perret et al., 2000; Spaink, 2000). Briefly, the bacteria recognize legumes secretions that passively diffuse across the bacterial membrane (Recourt et al., 1989) via a transcription factor (TF), which typically encoded by nodD. Perret et al. (2000) demonstrated that these Nod factors are primary determinants that decide which legumes will be able to nodulate. Therefore, it has been accepted that the different biovars and species of rhizobia generate a diverse range of Nod factors.

### INFECTION INITIATION

Nodule organogenesis begins by an exchange of signals between plant and bacteria, resulting in the curling and colonization of root hairs by rhizobia. Plant-derived membranes then form a tubular structure, called the “infection thread,” which guides bacteria to the site of meristematic activity in the root cortex and acts as an effective barrier to confine the bacteria. To analyze the first step of this series of events at the protein level in a time-course study with soybean over the first 48 h, Salavati et al. (2012a) combined 2-D gel electrophoresis coupled with quantitative RT-PCR to analyze isolated proteins at different time points from infected soybean root hairs at both transcriptional and translational levels. Analysis of 56 proteins revealed the differential expression of plant proteins associated with important events, such as metabolism, cell signaling, and disease/defense response. The formation of infected legume nodules capable of fixing nitrogen requires the bacteria to activate two plant programs: one leading to nodule morphogenesis and the other leading to nodule infection. Proteomic techniques were demonstrated that Nod factors can induce nodule morphogenesis, and this appears to occur as a consequence of modifying existing plant hormonal signaling systems, such as the cytokinin pathway (Relic et al., 1994; Hirsch et al., 1997). From a plant perspective, nodule morphogenesis is a critical stage because allowing bacterial entry gives rise to the potential for non-symbiotic bacteria to try to enter and take advantage of the plant (Oldroyd and Downie, 2008). Whereas, bacterial mutants lacking specific nod genes can induce some plant responses such as formation of arrested infections, root hair deformation, plant gene induction, calcium spiking, etc. It seems that producing the correct type of Nod factor by bacteria is critical for successful establishment of ITs (Ardourel et al., 1994; Walker and Downie, 2000; Oldroyd and Downie, 2004).

The growing IT must find this pre-infection thread structure because it allows the IT to join cells, to change from being in the intercellular space, and to promote changes in the direction of its growth. In addition to Nod factor specificity, surface and secretory proteins can also play important roles in infection (Spaink, 2000). The bacteria are budded off the end of the ITs before the plant-derived cell wall surrounds the IT. The endocytotic budding process results in the releasing of bacteria into the plant cell surrounded by a plant-made membrane (Ardourel et al., 1994). Then, bacteria differentiate and express nitrogen fixation-required genes (Fischer, 1994; Mesa et al., 2008). Proteome studies of nodule environment have explained that it is an extraordinary case of an interaction that includes specific nutrient uptake systems (Day et al., 2001; Djordjevic, 2004), a specialized electron transport chain (Ekman et al., 2008; Mastronunzio and Benson, 2010), and specific modifications by bacteria to their lipopolysaccharide surface (Lee et al., 2008). Thus, the symbiotic environment forms a unique type of extracellular biology interaction within the plant cells.

The root hairs provide a brilliant position for microbial development. To this aim, it seems that rhizobia have various mechanisms to affix to roots including surface polysaccharides and secretory/surface proteins (Matthysse and Kijne, 1998; Rodriguez-Navarro et al., 2007). Many factors such as pH, nitrogen concentrations (Jamet et al., 2007; Cardenas et al., 2008; Chang et al., 2009), and specific growth conditions (Khatoon et al., 2012) can affect on this contribution of different components. Santos et al. (2000) and Vargas Mdel et al. (2003) have revealed that several enzymes such as catalases and superoxide dismutases help rhizobia to survive at the oxidative stress. It has been proposed that plant-produced ROS are involved in cross-linking of glycoproteins in the matrix of the ITs (Brewin, 2004). Hence, the capability of bacteria to deal with extracellular oxidative stress, throughout the symbiosis, is obviously important.

## ATTACHMENT AND SECRETED PROTEINS

**FIGURE 1:**
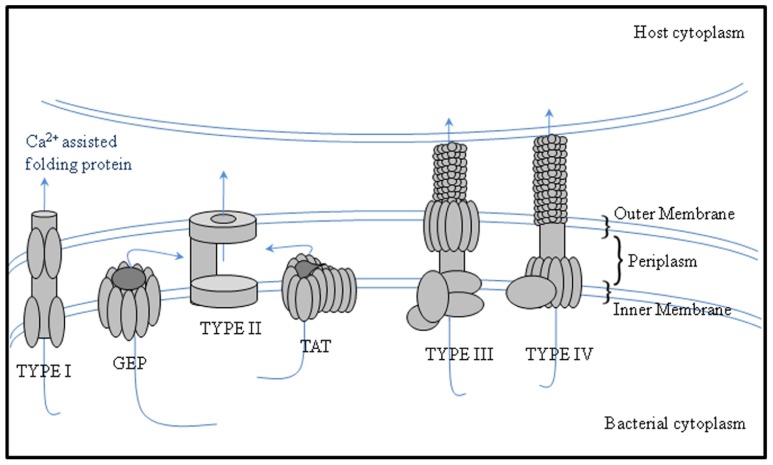
**Protein secretion systems secreting proteins to the periplasm or with periplasmic substrate intermediates in bacteria**. The schematic image of secretion systems in Gram-negative bacteria, which are involved in protein transport to the periplasm, are shown. Type I secretion systems translocate proteins from the periplasm, whereas Type II secretion systems translocate proteins from the periplasm and are thus dependent on the GEP or TAT pathways.

Secreted proteins are essential for attachment. For example, the role of rhicadhesin in attachment to root hairs has been described by Smit et al. (1992) and Laus et al. (2006). Rhizobium-adhering proteins (RAPs), which are encoded by the *prsD* and *prsE* genes (Russo et al., 2006), are secreted across the inner and outer membranes by means of a Type I secretion system. Different rhizobia have the potential to secrete proteins into the periplasm via the general export pathway and the twin-arginine translocation (TAT) secretion system and also type I, III, IV, and VI secretion systems that can manipulate the symbiosis (Deakin and Broughton, 2009; **Figure 1**; **Table 1**).

**Table 1:** Some important characteristics of protein translocation systems in Gram-negative bacteria.

	Secretion signal	Substrates	Reference
GEP	N-terminal signal peptide	Unfolded proteins	Paetzel etal. (2000)
TAT	N-terminal signal peptide with conserved twin-arginine motif	Fully folded proteins	Berks etal. (2003)
Type I	C-terminal signal peptide	Unfolded proteins	Wagner etal. (1983)
Type II	structural	Folded proteins or multimers	Pugsley (1993)
Type III	N-terminal signal	Unfolded proteins	Michiels etal. (1990)
Type IV	chaperone dependent	Folded or unfolded proteins or DNA	Berger and Christie (1994)
Type V	N-proximal secretion domain	Partly unfolded proteins	Henderson etal. (1998)

Many proteins of a typical N-terminal transit peptide are secreted via the general export pathway and many of these are expressed throughout infection and other steps of symbiosis. Cellulase (CelC2) is one of the particular symbiotic enzymes which is predicted to be exported through the general export pathway with a typical transit peptide (Downie, 2010). This cellulase can erode the non-crystalline cellulose in the cell wall of root hairs and is thought to allow penetration of rhizobia during the initial stage of infection in root hair curls (Robledo et al., 2008). Proteomics-based studies have demonstrated that proteins pertain to the TAT pathway are secreted in their folded form, some containing cofactors, and have a signal peptide usually containing a distinctive pair of consecutive twin arginine residues in the motif RRXFF, where X is any residue and F a hydrophobic residue (Antelmann et al., 2001; Bendtsen et al., 2005).

### TYPE I, III, IV, V, and VI SECRETION PATHWAYS

Proteins secreted via type I, III, IV, V, and VI secretion pathways, which translocated across the inner and outer membranes without a periplasmic intermediate stage (Hueck, 1998; Koronakis et al., 2004), are derived from the bacterial flagellar secretion system and have evolved into a mechanism that can deliver proteins from the bacterial cytoplasm into the cytoplasm of eukaryotic cells (Saier, 2004). Secreted proteins insert into the outer membrane, where it catalyses the translocation of the N-terminal region into the extracellular medium (Henderson et al., 2004) and mediates the translocation of proteins across the bacterial envelope (Bingle et al., 2008).

A cation selective channel protein, which is present in some rhizobia and can extend the ability to nodulation, is a calcium-binding protein called NodO (Economou et al., 1990; Sutton et al., 1994). NodO was found to insert into liposomes and form cation-selective channels in lipid bilayers (Miwa et al., 2006), and could therefore enhance the calcium spiking that is observed in root hairs upon Nod factor binding. Three possible roles for NodO have been suggested. The NodO protein may amplify the perceived Nod factor signal, facilitate Nod factor uptake by the host, or bypass the host’s Nod factor receptor altogether (Sutton et al., 1994; Walker and Downie, 2000).

The type III secreted proteins as well as the effector proteins delivered into the plant cell have been called nodulation outer protein (Nop; Viprey et al., 1998; Marie et al., 2003). By comparing plant defense responses against bacteria, Bent and Mackey (2007) showed that the mutilation of symbiotic efficiency might lead to effector recognition by legumes and subsequently, increasing at defense response. And some other effectors have been identified such as NopJ, NopM, NopL, NopP, NopT and ImpK. Some of which are required for optimal nodulation in legumes (Bladergroen et al., 2003; Marie et al., 2003). NopL and NopP are phosphorylated by plant kinases (Bartsev et al., 2003, 2004; Ausmees et al., 2004; Skorpil et al., 2005). This process could point to a function in modulating host signaling pathways. Specifically for NopL, a role in the down-regulation of host plant defenses is proposed (Bartsev et al., 2004). NopT is a functional cysteine protease of the YopT family with a predicted myristoylation site (Dai et al., 2008). Some type III secretory proteins such as NopT have protease activities (Kambara et al., 2009), and some proteins such as NopJ and NopM have negative effects on the nodulation (Marie et al., 2003). YopJ family-like proteins acts as acetyl transferases and inactivate MAP kinases and contain protein–protein interacting leucine-rich repeats (Deakin and Broughton, 2009; Masson-Boivin et al., 2009). XopD and YopM are targeted to host cell nuclei and were interfere with the regulation of host proteins during infection (Skrzypek et al., 1998; Hotson et al., 2003). XopD, a cysteine protease, achieves this through hydrolysis of small ubiquitin-like modifier-conjugated proteins (Hotson et al., 2003), while YopM probably acts as a scaffold for recruiting and stimulating other proteins (McDonald et al., 2003).

Autotransporter proteins (type V) are secreted via the general export pathway into the periplasm using an N-terminal transit peptide and then a C-terminal domain inserts into the outer membrane, where it catalyses the translocation of the N-terminal region into the extracellular medium (Henderson et al., 2004). Recently, type VI secretion system has been recognized, mediating the translocation of proteins across the bacterial envelope (Mougous et al., 2006; Pukatzki et al., 2006). Proteins translocated by this system were identified and showed similarity to ribose-binding proteins from other bacteria (Filloux et al., 2008; Pukatzki et al., 2009). Some bacterial substrates and their roles were summarized in **Table 2**.

**Table 2:** Examples of bacterial substrates and their roles.

Bacterial species	Substrate	Role	Secretion pathwa
*Agrobacterium rhizogenes*	GALLS	Integration of T-DNA into plant genome	IV
*Agrobacterium tumefaciens*	VirD2	Nuclear localization and integration of T-DNA	IV
*Bordetella pertussis*	AB5 toxin	Interacts with α β γ heterotrimeric Gi/o proteins	IV
*Bordetella *spp.	SphB1	Proteolytic processing of secreted proteins	V
	CyaA	Leukotoxin, adenylate cyclase	I
*E. coli*	AIDA-1	Adherence	V
	AG43	Biofilm formation	V
	Tsh	Haemoglobinase activity	V
	EspP	Cytotoxic activity	V
	Vat	Vacuolating cytotoxin	V
	HlyA	Haemolysin	I
*Erwinia *spp.	PrtB/C	Metalloproteases	I
	DspA/E	Suppresses cell wall defense responses	III
	HrpW	Binds to pectate lyase	III
*Haemophilus *spp.	HMW1	Adhesin	V
	HxuA	Haem-haemopexin binding protein	V
	LspA1	Adhesin	IV
*Helicobacter *spp.	CagA	Leads to dephosphorylation of host cell proteins	IV
	Peptidoglycan	Induces NF-κB activity in gastric epithelial cells	IV
*Legionella *spp.	RalF	Exchange factor for ADP ribosylation factor	IV
	LidA	Docking of vesicles to the membrane of phagosome	IV
	DotA	Membrane pore in host	IV
	LepA	Alter exocytic pathway in protozoa	IV
	LepB	Alter exocytic pathway in protozoa	IV
	YlfA	Facilitate binding and fusion of *Legionella*-containing vacuole	IV
	YlfB	Facilitate binding and fusion of *Legionella*-containing vacuole	IV
	VipA	affects carboxypeptidase trafficking	IV
	VipD	interferes with multivesicular body formation	IV
	VipF	inhibits lysosomal protein trafficking	IV
*Mesorhizobium *spp.	Msi059	Deconjugates proteins stabilized by SUMOylation	IV
	Msi061	Target specific proteins for degradation	IV
*Pasteurella* spp.	LktA/C	Leukotoxin	I
*Proteus *spp.	HpmA	Cytolysin	V
*Pseudomonas syringae*	AvrPto	Inhibits hypersensitive response (HR)	III
	AvrRpt2	Cysteine protease	III
	AvrRpm1	Induces RIN4 phosphorylation	III
	HopPtoM	Suppresses salicylic acid-dependent callose deposition	III
	AvrPphC	Blocks AvrPphF-elicited HR	III
*Rhizobium* spp.	NopP/L	Suppress plant defense reactions	III
	PlyB	Glycanase processing extracellular polysaccharide	I
	NodO	Facilitates nodulation	I
	. Rzc-1	Bacteriocin activity	I
*Salmonella *spp.	SipA/C	Enhance actin polymerization and bundling	III
	SopE/E2	Activate G-binding proteins	III
	SopB	Phosphatidylinositol phosphatase	III
*Serratia marcescens*	ShlA	Cytolysin	V
	SlaA S	Layer protein	I
	LipA	Lipase	I
	PrtA	Metalloprotease	I
	HasA	Haem-binding	I
*Shigella flexneri*	IcsA	Intracellular motility	V
	SigA	Cytopathic activity	V
*Xanthomonas *spp.	Hpa1/G	Elicits HR	III
	AvrXv4	Cleaves SUMO from sumoylated proteins	III
	XopD	Cleaves free and protein-bound SUMO	III
*Yersinia *spp.	YopH	Dephosphorylates Cas	III
	YopE	Activates signaling GTPases	III
	YopT	Cysteine protease	III
	YopO	Exhibits serine/threonine kinase activity	III
	YopJ	Cysteine protease	III
	YopM	Forms a complex with and activates kinases RSK1 and PRK2	III

*Type I secretion is completely independent of the general export pathway (GEP) and transports proteins directly from the cytoplasm across both membranes to the extracellular space. Type II secretion systems translocate proteins from the periplasm and are thus dependent on the GEP or TAT pathways. Type III systems translocate proteins across both membranes using ATP. Type IV systems is capable of transporting both DNA and proteins. This system is used to introduce the T-DNA portion of the Ti plasmid into the plant host. Type V secretion or autotransporters, is a simple system which the secreted protein transports itself across the outer membrane after being transported to the periplasm by the GEP.*

### NODULINS AND LEGHEMOGLOBIN

Nodulins are organ-specific plant proteins induced during SNF. Nodulins play both metabolic and structural roles within infected and uninfected nodule cells. Nodulins are involved in the structure, development, maintenance, and general metabolism of nodule (Oaks and Hirel, 1985) and have been characterized from soybean (Lauridsen et al., 1993), pea (van de Wiel et al., 1990), and alfalfa (Hirsch et al., 1989). Although characterized prior to other nodulins, the leghemoglobins (Lbs) should be considered as major nodulins. Soybean contains four major leghemoglobins, Lba, Lbcl, Lbc2, and Lbc3, differing slightly in amino acid sequence (Lee and Verma, 1984).

The nodulin-24, a protein which is part of the peribacteroid membrane (Katinakis and Verma, 1985) and nodulin-23, along with Lb, are induced in the infected cells. The nodulin-35, the subunit of the tissue-specific uricase II (a tetrameric enzyme) and one of the most abundant proteins in soybean nodules (Takane et al., 1997), is found only in the specialized uninfected cells. Formation of the effective root nodule requires expression of symbiotic genes in the host plant and the micro symbiont (Verma et al., 1986) whose products are nodulins and bacteroidins, respectively. Nodulin-24 is a nodule-specific and nodule-enhanced pbm-bound protein (Cheon et al., 1994), which has a transport function. The genes coding for Lb, nodulin-23 and -24, whose transcription begin at about the same time, provide a rationale for the possible existence of 5′ *cis* sequences capable of binding *trans*-activator molecules. During nodule development, some products elaborated from the microsymbiont infection that may produce a *trans*-activator molecule. The presence of short sequences common to the flanking region of the genes concerned may provide the structural basis for induction. In view of a potential *cis*-receptor site upstream of nodulin genes, it is possible that these genes are positively regulated by a common transactivator molecule.

In indeterminating nodule-forming legumes, the leading nitrogen transport compounds are amides including glutamine and asparagine, whereas in determinating nodule-forming legumes, the major nitrogen products are ureides. This process occurs in plastids of both infected and uninfected nodule cell types (Streeter, 1991; Vance, 2008). Enzymes concerned may be “nodule-stimulated” proteins. A group of enzymes for the oxidation of purines into allantoin and allantoic acid are specifically induced during symbiosis. Xanthine dehydrogenase is a nodulin present in infected nodule cells (Montalbini et al., 1997; Todd et al., 2006) and can catalyze these purine oxidation steps in the infected nodule cell. The product is postulated to be transported to uninfected cells as uric acid. The oxidation of uric acid to allantoin is mediated by the oxygen-dependent enzyme uricase II (nodulin-35).

### TRANSCRIPTION FACRORS

It is already obvious that TFs play fundamental roles in important processes in legumes and are involved in the control of mutualistic symbiosis such as SNF between plant root and rhizobia (Samac and Graham, 2007). More recently, TFs involved in the rhizobial infection process have been identified. Among them nodulation signaling pathway 1 (NSP1) and NSP2, GRAS-family proteins, are putative TFs that transduce the bacterial Nod factor signal and induce expression of plant nodulin genes and are required for nodule development (Kaló et al., 2005; Smit et al., 2005). In addition, other TFs crucial to the nodulation process were also identified by direct screening for nodulation-defective mutants. For example, NIN is the member of a novel family of putative TFs in higher plants, which are called the NIN-like family (Libault et al., 2009). Forward-genetics approaches have subsequently identified three other TFs (Nishimura et al., 2002; Mitra et al., 2004; Kaló et al., 2005; Smit et al., 2005) that are essential for nodule development, one of which is a Kruppel-like TFs of the C2H2 (Zn) family that was found to be crucial for differentiation of the nitrogen-fixing zone of nodules (Frugier et al., 2000). RNA interference (RNAi) method revealed a key role in nodule development for a member of the CCAAT-binding family of TFs (Combier et al., 2006).

### QUORUM-SENSING

The rhizobia are excellent quorum-sensing model systems for such studies. The symbiotic relationships are the result of a complicated signaling network between the host and symbiont. Rhizobia populations regulate gene expression by autoinducers, diffusible signal molecules, which are interact specially with a receptor protein. Autoinducers production occurs at specific stages of development or in response to environmental changes. These diffusible signals commonly induce gene expression in response to bacterial cell density in symbiosis and often referred as quorum-sensing (Bassler, 2002). However, we are not quite familiar with the regulation of differentiation of bacterial and host–bacterium signal exchange. It is believed that nodule invasion requires the gathering of bacteria around the hair roots and the cell density of *Rhizobium* species should reach a threshold level (Caetano-Anollés and Gresshoff, 1991). Therefore, quorum-sensing probably plays a curial function in regulation of symbiosis. In addition, some rhizobial strains are able to synthesize rhizopines, opine-like compounds reminiscent of those produced by *Agrobacterium* species, which are produced by the bacteroids and can be catabolized by the free-living associated rhizobia as a nutrient source (Murphy et al., 1987). These strains could putatively affect the dynamics of soil rhizobial populations.

In *R. leguminosarum* and *R. etli*, quorum-sensing probably is involved in restricting the number of nodules and in symbiosome development (Rosemeyer et al., 1998; Daniels et al., 2002; Wisniewski-Dye and Downie, 2002), and in *S. meliloti*, quorum-sensing controls the production of EPS II, an exopolysaccharide involved in the nodule invasion process (Marketon and González, 2002; Marketon et al., 2003). In different *Rhizobium* species, genes related to biosynthesis of exopolysaccharide, nodulation, and nitrogen fixation are located on one or more megaplasmids known as symbiotic (Sym) plasmids (Beynon et al., 1980; Banfalvi et al., 1985). Most of the nitrogen-fixing rhizobia regulate plasmid transfer via quorum-sensing systems, as in *A. tumefaciens.* While these systems do not seem to be essential for nodulation, they may play a role in rhizosphere survival (He et al., 2003). Although many aspects of the signal transduction are still an ambiguity, quorum-sensing has been intricate as an essential factor in the symbiotic process.

## CONCLUDING REMARKS AND FUTURE PERSPECTIVE

A considerable branch of the genome is devoted to the synthesis of the various proteins and the regulation of the interaction with their complex environment. While remarkable progress in proteomic study of symbiosis has been made in model plants, a quite advancement in developing proteomic approaches in other crops has been reached. The biggest obstruction to these proteomic applications is the scarcity of well-annotated protein databases and sequences of proteins. Although some techniques such as *de novo *sequencing and proteogenomics recompense this paucity, there is still an urgent need to expand and curate plant protein databases. Many existing databases, including Soybean, *Medicago *and rice proteome database should be expanded and integrated in the future (Komatsu et al., 2004; Sun et al., 2009). In addition, most proteomic studies lead to protein identification and functional predictions yet the majority do not test their results using genetic approaches. A combination of proteomic analysis with genetics and other omics approaches would intensify the biological significance of many studies (Kint et al., 2010; Salavati et al., 2012a). Furthermore, transcriptomics and analysis of strains carrying multiple mutations will help in future research. A more systematic integration of interdependent techniques would provide valuable information and leads to better prediction and management of plant responses to symbiotic bacteria.

On the one hand, up to 20% of net photosynthetic production would be used for production and maintenance of root nodules (Pate and Givnish, 1986). Therefore, legume can invest in nodules if a decrease in net photosynthesis is compensated by the nitrogen fixation (Bethlenfalvay et al., 1978). On the other hand, there is no guarantee that all inoculated rhizobia strains will ensure a net benefit because rhizobial strains have been demonstrated to vary considerably in the advantages that they provide to the legume (Thrall et al., 2007). If legumes could be able to discriminate between slightly and highly effective bacterial strains at the time of infection, there would be slight host carbohydrate loss to non-effective strains. Because, the nitrogen fixation begins a few days after inoculation, pre-infection recognition and exclusion of non-effective strains is inefficient.

Moreover, practical difficulties, such as high cost of performing field trails on Nod factor effects on nodulation, low stability of Nod factors in the soil due to quickly hydrolyzation in the rhizosphere by plant enzymes (Staehelin et al., 1994) and subsequently low biological activity of hydrolyzed derivatives (Heidstra et al., 1994) and mechanism of auto-regulation of nodulation (Caetano-Anollés and Gresshoff, 1991; Salavati et al., 2012a), lead to encountering the applied science to a new challenges which include discovering the bacterial and legume proteins, metabolites and their related genes effective on the signal production, perception, and transduction between partners. This proteomics strategy may open up an alternative perspective for improving symbiosis. Identification of genes and proteins and their related pathways may eventually lead to the production of new recombinant organisms, which have higher efficiency and ability to provide symbiosis and nitrogen fixation.

## Conflict of Interest Statement

The authors declare that the research was conducted in the absence of any commercial or financial relationships that could be construed as a potential conflict of interest.

## Abbreviations

GEP: the general export pathway
IT: infection thread
Lbs: leghemoglobins
LCOs: lipochitooligosaccharides
Nop: nodulation outer protein
NSP: nodulation signaling pathway
pbm: peribacteroid membrane
RAP: rhizobium-adhering proteins
RNAi: RNA interference
SNF: symbiotic nitrogen fixation
TAT: twin-arginine translocation pathway
TF: transcription factors.
